# Prognostic value from integrative analysis of transcription factors c-Jun and Fra-1 in oral squamous cell carcinoma: a multicenter cohort study

**DOI:** 10.1038/s41598-017-05106-5

**Published:** 2017-08-08

**Authors:** Hao Xu, Xin Jin, Yao Yuan, Peng Deng, Lu Jiang, Xin Zeng, Xiao-Song Li, Zhi-Yong Wang, Qian-Ming Chen

**Affiliations:** 1 Department of epidemiology and health statistics, West China School of Public Health, Sichuan University, Chengdu, 610041 China; 2 Department of Oral Biology and Medicine, State Key Laboratory of Oral Diseases, West China School of Stomatology, Sichuan University, Chengdu, 610041 China; 3 School of Mathematics, Sichuan University, Chengdu, 610041 China; 4 Chongqing Key Laboratory of Oral Diseases and Biomedical Sciences, College of Stomatology, Chongqing Medical University, Chongqing, 400016 China

## Abstract

Transcription factors c-Jun and Fra-1 have been reported to play a role during the initiation and progression in oral squamous cell carcinoma (OSCC). However, cohort studies are rarely reported. Here is an integrative analysis of their prognostic value in OSCC through a multicenter cohort study.313 OSCC patients were included in this study and received regular follow-up. The survival rate and hazard ratios(*HR*) were generated by survival analysis. The concordance probability and receiver operating characteristic curve area were chosen to measure the model discrimination. High expressions of c-Jun or Fra-1 were associated with poor prognosis, meanwhile the high expression of Fra-1 meant worse prognosis of patients than the high expression of c-Jun. Besides, the interaction effect of c-Jun and Fra-1 was antagonism, when the expression of c-Jun and Fra-1 was both high, the *HR* was lower than the hazard ratio when only the Fra-1 was at high expression. c-Jun and Fra-1 were both proved to be high risky predictors of death in OSCC, the antagonistic effect suggested that these biomarkers’ activities could be influenced by each other. It may provide a new sight for the studies of OSCC prognosis and treatment.

## Introduction

Oral squamous cell carcinoma (OSCC), a major devastating head and neck cancer subtype, is one of the most common cancers worldwide^1–3^. OSCC can migrate into the maxillary and mandibular bones and has a potent capacity to invade locally and metastasize distantly^4^. The five-year survival rate of patients with OSCC is less than 50%^5, 6^. In the clinical practice, the clinical tumor-node-metastasis (TNM) stage which includes tumor stage, lymph nodal stage and metastasis is usually used to predict the progression of OSCC^7, 8^. But the TNM staging system seems like the outcome of tumor prognosis instead of prediction, and patients with same TNM stages of OSCC may result in dramatically different survival time^7, 9, 10^. Hence, identification of novel and effective biomarkers is in need, which may serve as prognostic predictors, and also be used to guide treatment of OSCC patients^1^. To date, the application of gene-based biomarkers in diagnosis and prognosis of OSCC seem to be very promising^11–14^.

Activator protein 1 (AP-1) is one of the first identified transcription factors that regulates gene expression Signals like cytokines, hormone, infection and reactive oxygen species (ROS) can activate AP-1^15^, mainly through mitogen-activated protein kinase (MAPK)^16^. The activated AP-1 could increase the transcription of target genes and play roles in intercellular events including cell division, proliferation, differentiation, apoptosis and so on^17–19^. It is reported that the AP-1 was related to the tumorigenesis^20–22^, and the overexpression of c-Jun and Fra-1 promotes the invasive growth and metastasis of various tumors^23, 24^, such as breast cancer^24, 25^, liver cancer^26^, skin cancer^23^ and squamous-cell carcinoma^27, 28^, and indicated that they may be the potential therapeutic targets for SCC^28^.

AP1 is characterized as basic leucine-zipper domain, including Jun proteins (c-Jun, JunB, JunD), Fos proteins (c-Fos, FosB, Fra-1, Fra-2), activating transcription factor (ATF) proteins and musculoaponeurotic fibrosarcoma (MAF) proteins^16^. Most proteins which constitute the AP-1 belong to the JUN proteins, and among these, c-Jun is unique in regulating the cell proliferation^29, 30^. c-Jun exhibits the highest activation potential in the DNA binding affinities and transactivation capacities of the JUN proteins^15, 31^. FOS proteins are another main group of AP-1, which are just behind JUN proteins, it is best characterized as immediate early genes^16, 32^. Among the FOS proteins, apart from c-Fos, Fra-1 is best studied subunit of the Fos proteins, the transcriptional activity of Fra-1 is regulated both transcriptionally and post translationally^33, 34^. However, current studies explained the influences of c-Jun and Fra-1 on OSCC mainly with experimental models^35–38^. The hazard risks of c-Jun and Fra-1 overexpression in the OSCC prognosis and the correlations between them and cellular biological processes in OSCC with tissue microarray (TMA) which derived from multicenter cohort study are rarely reported.

Thus, in this study, we focused on the two subunits of AP-1, c-Jun and Fra-1. We performed an integrative analysis of the association between the two subunits of AP-1 and the prognosis of OSCC with multicenter cohort study. And as the intricate relationship of the AP-1 subunits on the tumorigenesis and tumor prognosis^15, 39^, the interaction effect of c-Jun and Fra-1 on the prognosis was also investigated.

## Materials and Methods

### Patients’ cohorts

The West China Hospital of Stomatology (Chengdu, China), Guangdong Provincial Stomatological Hospital (Guangzhou, China) and the General Hospital of the People’s Liberation Army (Beijing, China) participated this study. The study was approved by the ethics committees of all the three hospitals, and was conducted in agreement with the Helsinki Declaration. Written informed consent was provided by all participants at baseline and during follow-up.

A total of 313 postoperative patients from three hospitals with primary OSCC tumors constituted this multicenter cohort study and received regular follow-up. Beside the regular visits, all patients could initiate follow-up visits if they were concerned that they had recurrence or a new primary tumor. Information was collected during the follow-up visits, which included the medical history and clinical examination, such as age, gender, smoking status, drinking status, tumor differentiation, clinical TNM stage and primary site of tumor. The survival time of each patient was recorded from the day of surgery until the time of cancer-related death or the end of the follow-up period (5 years), death for other reasons led to censoring of data.

### Immunohistochemistry

For immunohistochemical analysis of c-JUN and Fra-1, tissue microarray (TMA) slides of the patients from the three hospitals were used, containing 313 evaluable samples from formalin fixed, paraffin-embedded OSCC cases. EnVision system was used on the staining, described as previous studies^40^. The results were reviewed by two pathologists independently, and the discrepancies in immunostaining reviewing were solved by consensus. The stained slides were scanned using an Aperio Scanscope (Aperio, USA) and quantified with the available Aperio algorithms^41^, the original immunohistochemical staining of patients’ tissues the West China Hospital of Stomatology were showed in Supplementary Fig. S1. The immunostaining results of c-Jun and Fra-1 could be divided into high expression and low expression according to percent of cells stained and staining intensity^8^. The intensity of staining was scored as follows: 0, no color; 1, light yellow; 2, light brown; 3, brown. The number of positive cells was scored as follows: 0, <5%; 1, 5–25%; 2, 25–50%; 3, >50%. The two grades were multiplied together, producing scores from 0 to 9 that were classified as follows: weak staining (0–4 scores); strong staining (6–9 scores).

### Statistical analysis

The differences in expression levels among different baseline characteristics of the patients were detected by the *t* test for continuous variables, the *χ* ^*2*^ test or Fisher’s exact test for categorical variables, the Kruskal-Wallis *H* test for ordinal variables. The correlation between the expression of c-Jun and Fra-1 was explored by Kendall’s *tau*. Overall survival (OS) at 5 years was evaluated by the Kaplan–Meier method, with the log-rank test in the univariate analysis between high and low expression of c-Jun and Fra-1. Multivariate survival analysis was performed with the Cox proportional hazards model, and the interaction effect of Fra-1 and c-Jun was tested, the Hazard Ratio (*HR*) of Cox model was used for evaluate the survival risk, which is the ratio of the hazard rates corresponding to the conditions described by two levels of an explanatory variable^42^. The concordance probability and receiver operating characteristic (ROC) curve area were chosen to validate the Cox model discrimination among different models with different independent variables^43–45^. Statistical analyses were performed in R packages (version 3.1.2), mostly with the “survival” package^46^. Unless stated otherwise, two-sided significance level was 0.05.

## Results

### Demographic characteristics

**Figure 1:**
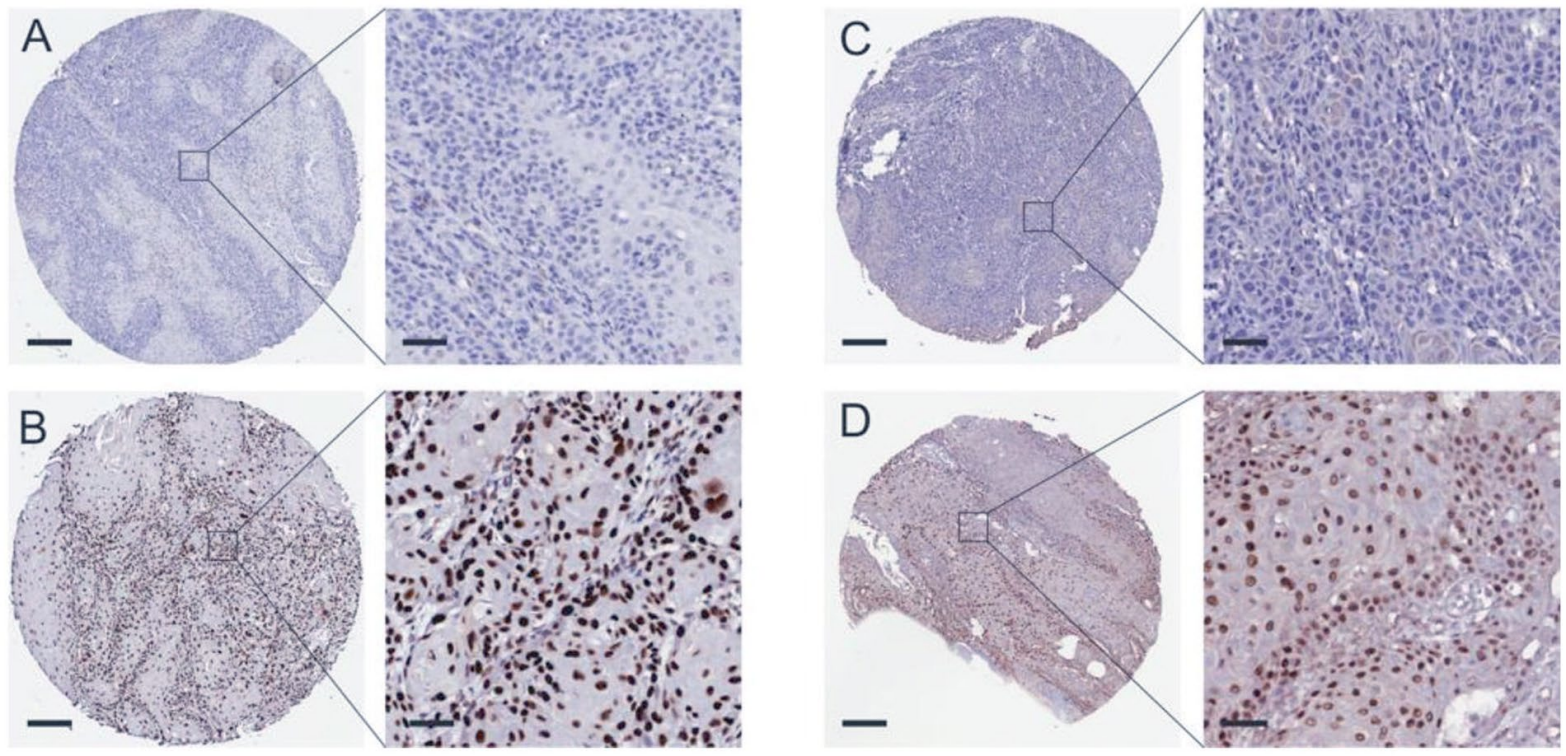
The immunohistochemical staining of c-Jun and Fra-1. Performed by EnVision system, protein immunoreactive substances were mainly displayed in the nucleus. (**A**) Low expression of c-Jun. Black scale bars at the bottom represent 200 µm (left side) or 50 µm (right side); (**B**) high expression of c-Jun; (**C**) low expression of Fra-1; (**D**) high expression of Fra-1.

The median follow-up time of these OSCC patients was 21 months. 69% of these patients were male, which are twice as much as female. About 50 percent of them were over 60 years old. The immunostaining showed that most tumor cells had a very bright nuclear positive c-Jun expression, a nuclear and cytoplasm positive Fra-1 expression (Fig. 1). High level of c-Jun was detected in 72.5% of the patients, and high level of Fra-1 was detected in 57.2% of the patients from multi centers.

### Associations between the immunostaining results and the patients’ characteristics

Next, we evaluated the association of expression levels of two AP-1 subunits and other characteristics in OSCC. The results of *χ* ^*2*^ test, Fisher’s exact test and Kruskal-Wallis *H* test, showed that the expression status of c-Jun and Fra-1 varies among different tumor stages, nodal stages or clinical TNM stage (Table 1), and the differences were statistically significant. We also noticed that there was a trend that the association between and the tumor clinical TNM stage and the expressions of c-Jun and Fra-1 was positive, which showed that the higher TNM stages’ patients had higher level of the two protein expressions. In the contrary, gender, age, smoking status, drinking status, primary sites of OSCC and cell differentiation are not associated with expression of c-Jun and Fra-1 at the 5% significance level.

**Table 1:** The association between the expression of c-Jun and Fra-1 with the baseline characteristics of the patients with OSCC.

		Total (100%)	c-Jun		*P value*	Fra1		P value*
			low expression	high expression		low expression	high expression	
**NO. patients (all)**		**313 (100)**	**86 (27.5)**	**227 (72.5)**		**134 (42.8)**	**179 (57.2)**	
gender	male	213 (68.05)	57 (26.76)	156 (73.24)	0.598	89 (41.78)	124 (58.22)	0.635
	female	100 (31.95)	29 (29)	71 (71.00)		45 (45.00)	55 (55.00)	
age	≤60 yr	159 (50.80)	48 (30.19)	111 (69.81)	0.213	70 (44.03)	89 (55.97)	0.596
	>60 yr	154 (49.20)	38 (24.68)	116 (75.32)		64 (41.56)	90 (58.44)	
smoking	never	167 (53.35)	50 (29.94)	117 (70.06)	0.303	72 (43.11)	95 (56.89)	0.976
	ever	146 (46.65)	36 (24.66)	110 (75.34)		62 (42.47)	84 (57.53)	
drinking	never	182 (58.15)	53 (29.12)	129 (70.88)	0.521	78 (42.86)	104 (57.14)	0.917
	ever	131 (41.85)	33 (25.19)	98 (74.81)		56 (42.75)	75 (57.25)	
primary site	cheek	52 (16.61)	13 (25.00)	39 (75.00)	0.54	20 (38.46)	32 (61.54)	0.145
	tongue	114 (36.42)	35 (30.70)	79 (69.30)		60 (52.63)	54 (47.37)	
	gum	66 (21.09)	15 (22.73)	51 (77.27)		26 (39.39)	40 (60.61)	
	others	81 (25.88)	23 (28.4)	58 (71.60)		28 (34.57)	53 (65.43)	
cell differentiation	high	193 (61.34)	56 (29.17)	137 (70.83)	0.696	83 (42.71)	110 (57.29)	0.921
	moderate	92 (29.39)	22 (23.91)	70 (76.09)		39 (42.39)	53 (57.61)	
	low	28 (8.95)	8 (28.57)	20 (71.43)		12 (42.86)	16 (57.14)	
tumor stage	T1	45 (14.38)	17 (37.78)	28 (62.22)	**0**.**002**	22 (48.89)	23 (51.11)	**0**.**029**
	T2	139 (44.41)	48 (34.53)	91 (65.47)		68 (48.92)	71 (51.08)	
	T3	61 (19.49)	9 (14.75)	52 (85.25)		24 (39.34)	37 (60.66)	
	T4	68 (21.73)	12 (17.65)	56 (82.35)		20 (29.41)	48 (70.59)	
nodal stage	N0	168 (53.67)	57 (33.93)	111 (66.07)	**0**.**011**	81 (48.21)	87 (51.79)	**0**.**023**
	N1-3	145 (46.33)	29 (20)	116 (80)		53 (36.55)	92 (63.45)	
clinical TMN stage	I	37 (11.82)	18 (48.65)	19 (51.35)	**0**.**003**	24 (64.86)	13 (35.14)	**0**.**001**
	II	94 (30.03)	30 (31.91)	64 (68.09)		46 (48.94)	48 (51.06)	
	III	127 (40.58)	30 (23.62)	97 (76.38)		52 (40.94)	75 (59.06)	
	IV	55 (17.57)	8 (14.55)	47 (85.45)		12 (21.82)	43 (78.18)	

^*^The p value was generated using χ^2^ test, Fisher’s exact test and Kruskal-Wallis H test.

As appeared in Table 1, the distribution trend of c-Jun expression among different population characteristics was similar with Fra-1. The Kendall’s *tau* was calculated for evaluating the correlation between the expression of c-Jun and Fra-1. And the *tau* was 0.313 (*P* < 0.001), indicated that there was a positive correlation between the expression of c-Jun and Fra-1 in the OSCC.

### Univariate analysis of the patients’ overall survival

The overall survival at 5 years was 0.345 (95%*CI*: 0.286, 0.416) in the multicenter cohort, and different groups of cohorts owned different 5 years OS, but some of them had no statistical significances (*P* > 0.05 under the log-rank test), as shown in Table 2. The 5 years OS of the group without lymphatic metastasis (0.451, 95%*CI*: 0.364, 0.560) was higher than in the group with lymphatic metastasis (0.238, 95%*CI*: 0.171, 0.332, log-rank test *P* < 0.001). And from the Table 2 we could also see that the groups with lower tumor clinical TMN stage had the higher 5 years OS than the groups with higher tumor clinical TMN stage (*P* = 0.001). The 5 years OS values in different groups of genders, age groups, smoking status, drinking status, primary sites of tumor, cell differentiation or tumor stages had no statistical differences.

**Table 2:** The Overall Survival (OS) at 5 years in each subgroup population with OSCC.

		OS at 5 years (95%CI)	*p* value*
gender	male	0.326 (0.257, 0.413)	0.547
	female	0.384 (0.283, 0.52)	
age	≤60 yr	0.298 (0.221, 0.402)	0.851
	>60 yr	0.402 (0.319, 0.505)	
smoking	never	0.339 (0.266, 0.431)	0.624
	ever	0.349 (0.258, 0.473)	
drinking	never	0.336 (0.261, 0.433)	0.742
	ever	0.355 (0.267, 0.471)	
primary site	cheek	0.408 (0.262, 0.637)	0.176
	tongue	0.33 (0.232, 0.471)	
	gum	0.331 (0.223, 0.492)	
	others	0.318 (0.224, 0.451)	
cell differentiation	high	0.336 (0.264, 0.429)	0.33
	moderate	0.408 (0.298, 0.558)	
	low	0.252 (0.13, 0.49)	
tumor stage	T1	0.397 (0.26, 0.606)	0.068
	T2	0.399 (0.311, 0.512)	
	T3	0.295 (0.179, 0.486)	
	T4	0.243 (0.145, 0.409)	
nodal stage	N0	0.451 (0.364, 0.56)	<0.001
	N1-3	0.238 (0.171, 0.332)	
clinical TMN stage	I	0.58 (0.425, 0.791)	0.001
	II	0.413 (0.301, 0.566)	
	III	0.26 (0.181, 0.375)	
	IV	0.259 (0.155, 0.434)	
c-Jun	Low expression	0.555 (0.445, 0.693)	<0.001
	High expression	0.258 (0.193, 0.345)	
Fra-1	Low expression	0.543 (0.45, 0.655)	<0.001
	High expression	0.191 (0.13, 0.282)	

^*^ *p* value was generated using log-rank test.

**Figure 2:**
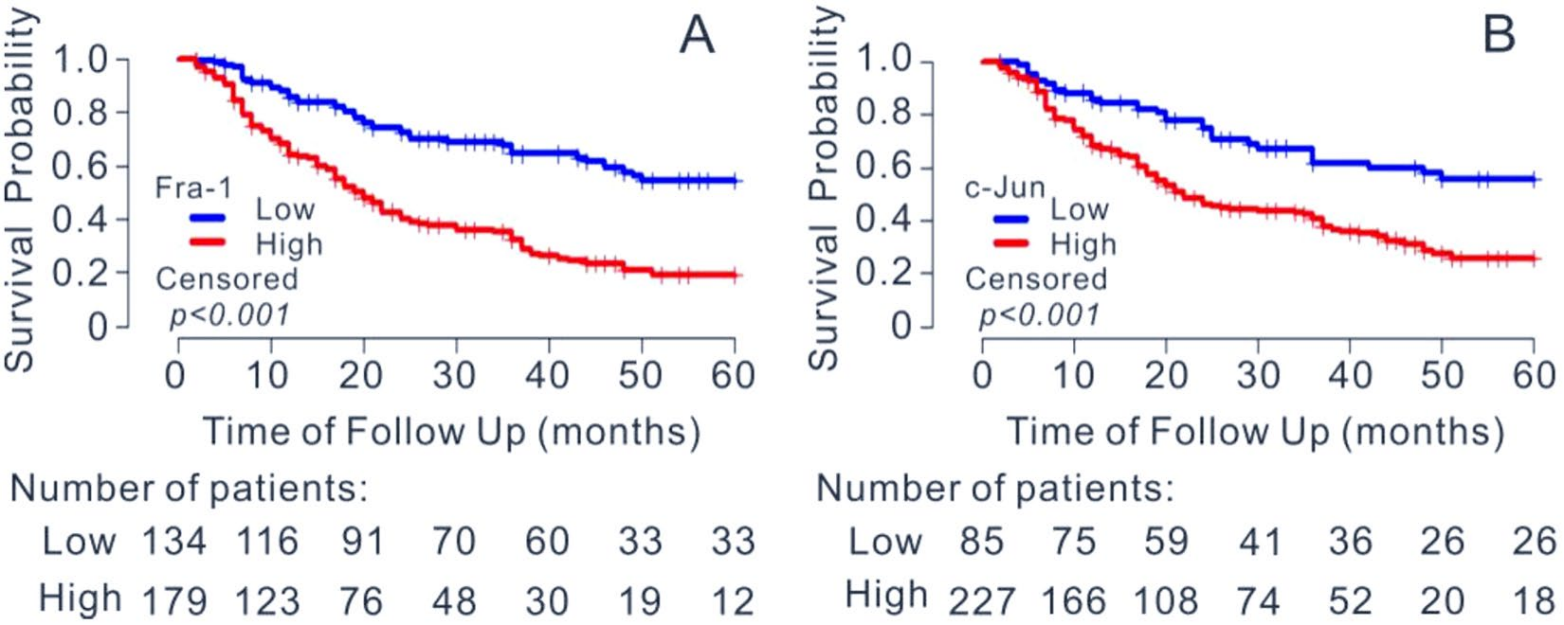
Survival curves of OSCC patients with different expression of c-Jun and Fra-1. (**A**) Correlation between 5-yr survival rate with the expression of Fra-1. (**B**) Correlation between 5-yr survival rate with the expression of c-Jun. The survival curves were defined by the Kaplan–Meier method, the tests of survival rates were performed by log-rank test.

The 5 years OS value (0.258, 95%*CI*: 0.193, 0.345) in the group with high level expression of c-Jun was lower than the value (0.555, 95%*CI*: 0.445, 0.693) in the group with low level expression (*P* < 0.001). Contrasted with the group with low expression of c-Jun, the survival risk of the high level expression group is higher, which the *HR* of high level expression is 2.295(95%*CI*: 1.552, 3.394). Analogously, the group with high level expression of Fra-1 owned a lower 5 years OS value than the group with the low-level expression, the *HR* of high level expression was 2.789 (95% *CI*: 1.994, 3.899) contrasted with the low expression of Fra-1, which mean higher survival risk. The survival curves of OSCC patients with different expression of c-Jun and Fra-1 were showed in Fig. 2 (the survival curves of OSCC patients separated by tumor clinical TNM stages could be found in Supplementary Fig. S2).

### Multivariate analysis of patients’ overall survival

To evaluate the influence of c-Jun and Fra-1 on the 5 years OS, multivariate Cox models were performed which were adjusted for demographic and clinical characteristics. One of them was considered the interaction effect of c-Jun and Fra-1, results were provided in Table 3. In the model without interaction factors, the group with high expression of Fra-1 had a poor prognosis compared to the group with low level expression of Fra-1, and the *HR* was 2.424 (95%CI: 1.651, 3.559). However, there was no statistical significance in the effect of c-Jun (*P* = 0.092). Since both c-Jun and Fra-1 are the subunits of the AP-1, we next evaluated the interaction effect of c-Jun and Fra-1. The model with interaction factor showed that the high expression of c-Jun and Fra-1 resulted in higher risk of death, and the *HR*s were 2.511(95%CI: 1.345, 4.688) and 5.625(95%CI: 2.685, 11.788) respectively, suggesting that both factors could promote progression OSCC. And the interaction effect between c-Jun and Fra-1 was 0.333 (95%CI: 0.144, 0.769), suggesting that the effect of c-Jun on the prognosis of OSCC could be obstructed by Fra-1, and vice versa (the interaction prognostic effects between c-Jun and Fra-1 during each clinical stage were showed in Supplementary Fig. S3). When the expression of c-Jun and Fra-1 was both high, the hazard risk of them was 4.703(2.511* 5.625 * 0.333), the interaction effect of c-Jun and Fra-1 was antagonism. The differences in the effects of c-Jun and Fra-1 between the Cox model without interaction factor and the Cox model with the interaction factor may be caused by the interaction effect between the c-Jun and Fra-1. The results in the model with the interaction factor would be chosen as it was comprehensive.

**Table 3:** Multivariate analysis of OSCC patients’ overall survival at 5 years.

		Multivariate Cox		Multivariate Cox with interaction	
		*HR* (95%CI)	*P* value*	*HR* (95%CI)	*P* value*
c-Jun	Low expression	1 ref	0.092	1 ref	0.004
	High expression	1.463 (0.940, 2.278)		2.511 (1.345, 4.688)	
Fra-1	Low expression	1 ref	<0.001	1 ref	<0.001
	High expression	2.424 (1.651, 3.559)		5.625 (2.685, 11.788)	
c-Jun*Fra-1	at least one is low	—		1 ref	0.01
	both are high	—		0.333 (0.144, 0.769)	
gender	male	1 ref	0.652	1 ref	0.629
	female	0.905 (0.585, 1.398)		0.898 (0.58, 1.39)	
age	≤60 yr	1 ref	0.881	1 ref	0.816
	>60 yr	0.976 (0.708, 1.345)		0.963 (0.699, 1.325)	
smoking	never	1 ref	0.634	1 ref	0.434
	ever	0.906 (0.603, 1.360)		0.85 (0.566, 1.277)	
drinking	never	1 ref	0.39	1 ref	0.462
	ever	0.828 (0.539, 1.273)		0.849 (0.55, 1.312)	
primary site	cheek	1 ref		1 ref	
	tongue	1.906 (1.144, 3.176)	0.013	2.018 (1.211, 3.364)	0.007
	gum	1.517 (0.865, 2.659)	0.146	1.662 (0.945, 2.924)	0.078
	others	1.740 (1.042, 2.905)	0.034	1.847 (1.104, 3.091)	0.019
cell differentiation	high	1 ref		1 ref	
	moderate	0.832 (0.565, 1.224)	0.35	0.843 (0.573, 1.242)	0.388
	low	1.338 (0.791, 2.266)	0.278	1.437 (0.844, 2.445)	0.182
tumor stage	T1	1 ref		1 ref	
	T2	0.742 (0.404, 1.363)	0.336	0.8 (0.431, 1.483)	0.478
	T3	0.661 (0.333, 1.312)	0.236	0.725 (0.36, 1.458)	0.367
	T4	0.998 (0.495, 2.011)	0.995	1.021 (0.503, 2.073)	0.955
nodal stage	N0	1 ref	0.018	1 ref	0.018
	N1-3	2.012 (1.128, 3.590)		2.015 (1.128, 3.599)	
clinical TMN stage	I	1 ref		1 ref	
	II	1.815 (0.814, 4.045)	0.145	1.677 (0.743, 3.782)	0.213
	III	1.382 (0.551, 3.462)	0.49	1.268 (0.502, 3.207)	0.616
	IV	1.084 (0.382, 3.078)	0.879	1.029 (0.357, 2.965)	0.958

However, the other factors in the models were not influenced by the interaction effect, the effects of the other factors which consisted by the demographic and clinical characteristics were all similarity between the two models. The primary sites of tumor and the lymphatic metastasis were another two factors with statistical significance on the prognosis of OSCC among the demographic and clinical characteristics. The patients were more likely to die if their primary sites of tumor were tongue and others, contrasted with the group with the primary site of tumor in cheek, the *HR*s were 2.018(95%*CI*: 1.211, 3.364) and 1.847(95%*CI*: 1.104, 3.091) in the model with interaction factor, respectively. The lymphatic metastasis could also lead to the worse prognosis, and the *HR* was 2.012(95%*CI*: 1.128, 3.590).

### Evaluation of the prognostic effects of c-Jun and Fra-1

**Figure 3:**
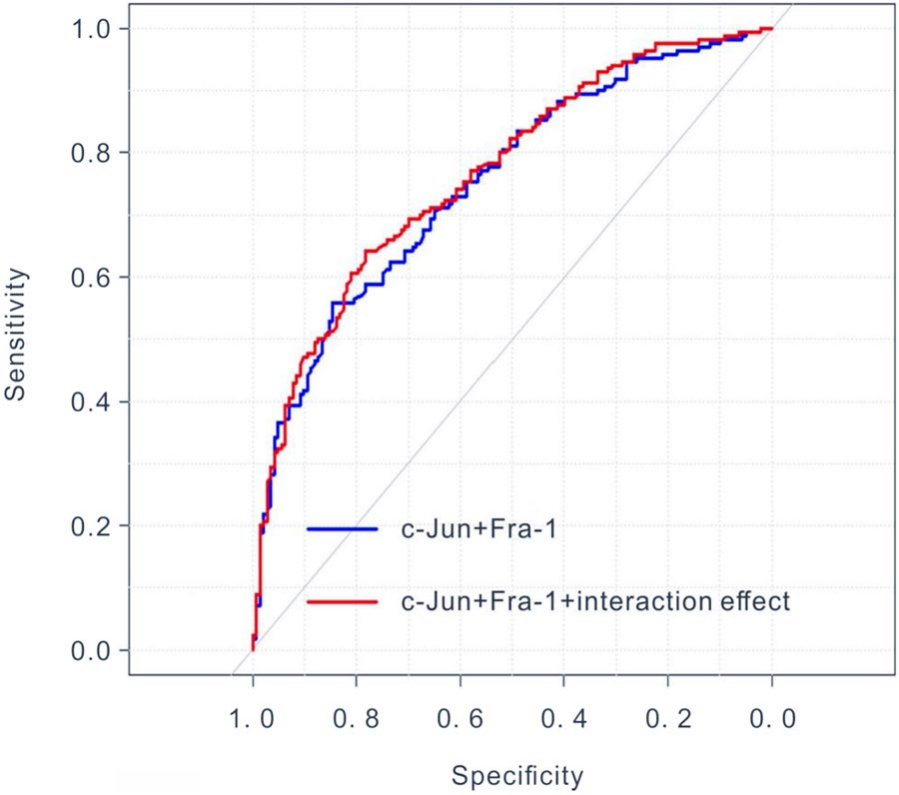
ROC curves for overall survival of OSCC patients with or without interaction effect of c-Jun and Fra-1.

Two multivariate Cox models were performed to estimate the effects of c-Jun and Fra-1 on the 5 years OS with adjusting for the confounding bias. To evaluate the predictive value of the two proteins, concordance probability and ROC were used to assess the discriminatory power and the predictive value of the two Cox models, especially the model with interaction factor. The higher concordance probability and the area under the ROC (AUC) of the model, the higher discriminatory power and predictive value of the model would be, and the higher predictive value on the prognosis of c-Jun and Fra-1 would be. The concordance probability of the Cox model without or with interaction factor were 0.689(95%*CI*: 0.641, 0.737) and 0.699(95%*CI*: 0.651, 0.747), and the ROC plots were showed in Fig. 3, the AUC of the model without interaction factor was 0.752(95%*CI*: 0.699, 0.805), and the other was 0.766(95%*CI*: 0.742, 0.818), they were all higher than 0.5. So the Cox models with c-Jun and Fra-1 owned high predictive value of the prognosis of OSCC. Besides, the concordance probability and AUC of the model with interaction factor were both higher than the values of the model without interaction factor, even though there was no statistical significance(*P* > 0.05). It suggested that the Cox model considered the interaction effect of c-Jun and Fra-1 was more valued than the model without interaction factor. the interaction factor which consisted by c-Jun and Fra-1, played an important role in the prognosis of OSCC, the same as the c-Jun and Fra-1.

## Discussion

AP-1 is involved in a wide range of cellular events, such as cell growth, proliferation, differentiation and apoptosis^16, 17^. It consists of various dimers of either homodimers or heterodimers. c-Jun and Fra-1 are the two most important subunits of AP-1. and c-Jun can regulate the cell processes as homodimers or heterodimers, but Fra1 needs to combine with JUN proteins to be functional^29, 33^. They both are reported to be cancer promoters^20^. However, few studies were focused on the associations between the two proteins and survival prognosis of OSCC with multi cohort study. This study was designed to explore the prognostic value of the two proteins on the survival prognosis of OSCC patients, and detect the interaction effect between c-Jun and Fra-1, by following up three cohorts in China. Three cohorts included 313 postoperative patients, were respectively located in the north, west and south of China, which could minimize the selection bias of patients.

According to the log-rank test, the population with high expression level of c-Jun or Fra-1 own lower survival rates. The demographic characters and clinical characters of patients were adjusted in the Cox model to reduce the confounding bias, such as gender, age, and smoking status, drinking status, tumor differentiation, clinical TNM stage and the primary site of tumor. Several Cox models have been made with different explanatory variables, after that AUC and concordance probability of the Cox model were used to establish the best model, which also help us to find the significance of the interaction effect between c-Jun and Fra-1^45, 47^. From the statistical analysis, the population of OSCC with high expression levels of the two proteins have worse survival prognosis, the hazard of survival in the population with high expression of c-Jun and Fra-1 were all higher than the low expression. It indicated that the c-Jun and Fra-1 are both valuable prognostic biomarkers in OSCC. However, the Cox model showed that the *HR* of high expression of c-Jun in the model is lower than the Fra-1, which suggested the high expression of Fra-1 would result in worse prognosis than the high expression of c-Jun in OSCC. It differs from *Robert Eferl* (2003)^20^, who reported c-Jun may have stronger transforming activity, our study indicates that Fra-1 may play a more important role in OSCC.

Besides, the AUC and concordance probability confirmed the model with interaction factor is the best predictive and discrimination model in this study, which means the interaction effect of c-Jun and Fra-1 exist truly in OSCC. And the interaction effect is antagonism, the hazard in the situation which c-Jun and Fra-1 are both at the high expression is lower than situation which only Fra-1 is at high expression for the OSCC patients. To date, the antagonistic prognostic effect of c-Jun and Fra-1 on OSCC patients has not been reported in other studies, as tumorigenesis of c-Jun and Fra-1 is not fully understood, the mechanism of this antagonism is still unclear. Maybe they are competing for binding to the AP-1 sites or by forming “inactive” heterodimers when they are all at high expression^15, 39^ and the cellular biological process of c-Jun and Fra-1 are complex, they may be also influenced by tumor or tissue types^20, 48, 49^.

The classification of the TMA staining was based on the percent of positive cells and staining intensity in our study, while c-Jun and Fra-1 also present in cytoplasm, for example, in certain types of cancers including breast, lung and thyroid cancer cytoplasmic Fra-1 over-expression has been reported, and there is also evidence showing that Fra-1 and c-Fos support growth of human malignant breast tumors by activating membrane biogenesis at the cytoplasm^50^. Specifically, in HNSCC, Serewko *et al*. describes Fra-1 expressed predominantly in nuclear^51^, similar in our study, c-Jun and Fra-1 were mainly appeared in the nucleus. As dispute exists^37^, it would be necessary to present the cytoplasm and nuclear expressions of these two proteins in HNSCC and their correlations with OSCC patients’ survival in the future study.

To further validated the results displayed by this study, studies with larger sample size of OSCC patients in different counties are needed. And to fully understand the mechanisms of possible antagonistic effect between c-JUN and Fra-1, functional evidences are also needed, more *in vivo* and *in vitro* experimental studies should be conducted. And it will be valuable to perform the longitudinal study to explore whether the expression of these proteins would be changed during the tumor progression.

In summary, c-Jun and Fra-1 could be another two valuable prognostic biomarkers in OSCC, and they may help to know more about the prognosis of OSCC. Meanwhile, we could try to find some new diagnostic methods and treatments through these two biomarkers. Besides, this study also indicated that the transforming activity of the AP-1 subunits could be influenced by each other, the interaction of tumor biomarkers may provide a new sight for the studies of tumor prognosis and tumor treatment in OSCC^22, 28^.

## Electronic supplementary material

Supplementary Figures
