# Supplementary material for: Prognostic value from integrative analysis of transcription factors c-Jun and Fra-1 in oral squamous cell carcinoma: a multicenter cohort study
Source: Sci Rep. 2017 Aug 8;7:7522. doi: 10.1038/s41598-017-05106-5 (PMC5548725; doi:10.1038/s41598-017-05106-5)

Prognostic value from integrative analysis of transcription factors c-Jun and Fra-1 in oral squamous cell carcinoma: a multicenter cohort study

Hao Xu[1,2,3#], Xin Jin[4,2#], Yao Yuan[2], Peng Deng[2], Lu Jiang[2], Xin Zeng[2], Xiao-Song Li[1], Zhi-Yong Wang[2*], Qian-Ming Chen[1,2*].

[1] Department of epidemiology and health statistics, West China School of Public Health, Sichuan University, Chengdu 610041, China;

[2] Department of Oral Biology and Medicine, State Key Laboratory of Oral Diseases, West China School of Stomatology, Sichuan University, Chengdu 610041, China;

[3] School of Mathematics, Sichuan University, Chengdu 610041, China;

[4] Chongqing Key Laboratory of Oral Diseases and Biomedical Sciences, College of Stomatology, Chongqing Medical University, Chongqing 400016, China.

# These authors contributed equally to this work

*** Corresponding author:**

Qian-Ming Chen, Department of epidemiology and health statistics, West China School of Public Health, Sichuan University, 16 South Renmin Road, Chengdu 610041, China; Fax & Phone: +86 28 85405251; E-mail: qmchen@scu.edu.cn.

Zhi-Yong Wang, Department of Oral Biology and Medicine, State Key Laboratory of Oral Diseases, West China School of Stomatology, Sichuan University, 14 South Renmin Road, Chengdu 610041, China; Phone: +1 3019066811; E-mail: zhiyong.wang@scu.edu.cn.

Titles and legends for Supplementary Figures

Supplementary Figure S1. The original immunohistochemical staining of patients' tissue from the West China Hospital of Stomatology (Chengdu, China)

A. The immunohistochemical staining of c-Jun.

B. The immunohistochemical staining of Fra-1

Supplementary Figure S2. The survival curves of OSCC patients with different expressions of c-Jun and Fra-1 in different tumor clinical TNM stages

The four images above were from c-Jun's (A, B, C, D) and the below were from Fra-1 (E, F, G, H). From left to right, they were stage 1 (A, E), stage 2 (B, F), stage 3 (C, G) and stage 4 (D, H), separately. The *P* values were from the log-rank tests for the overall survival.

Supplementary Figure S3. Interaction effects between c-Jun and Fra-1 during each clinical TNM stage

The points are the adjusted Hazzard Ratios (*HRs*), and the error bars are the 95% *CI*. The *HR* were from the interaction factors of c-Jun and Fra-1 in the multiple Cox models. The adjusted *HRs* (95%*CIs; P* values) are as followed: stage 1:172037(0, Inf.; *P*:0.9991); stage 2:0.1098 (0.0251, 0.4810; *P*:0.0034), stage 3:0.4056 (0.1164, 1.4140; *P*:0.1567), stage 4:1.1467 (0.0477, 27.5850; *P*:0.9328).

Supplementary Figure S1.

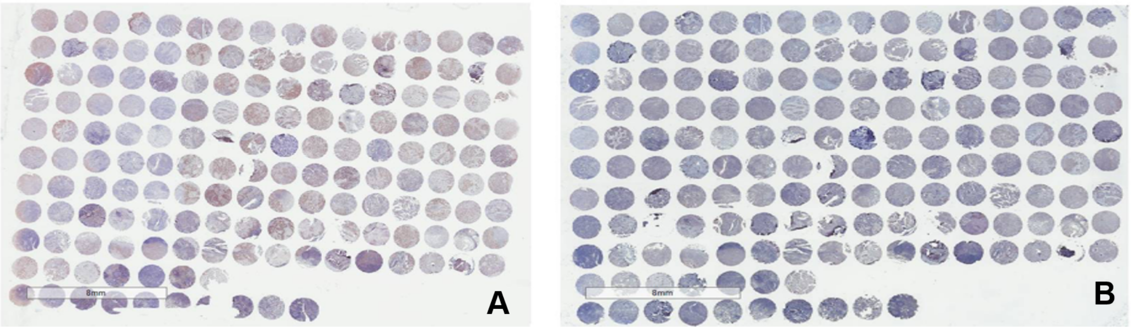

Supplementary Figure S2.

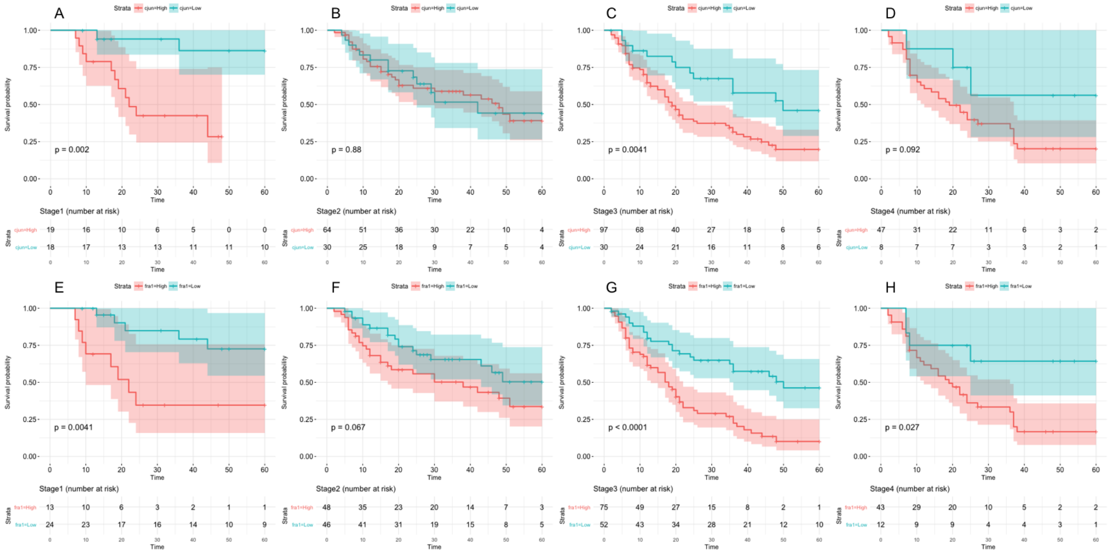

Supplementary Figure S3.

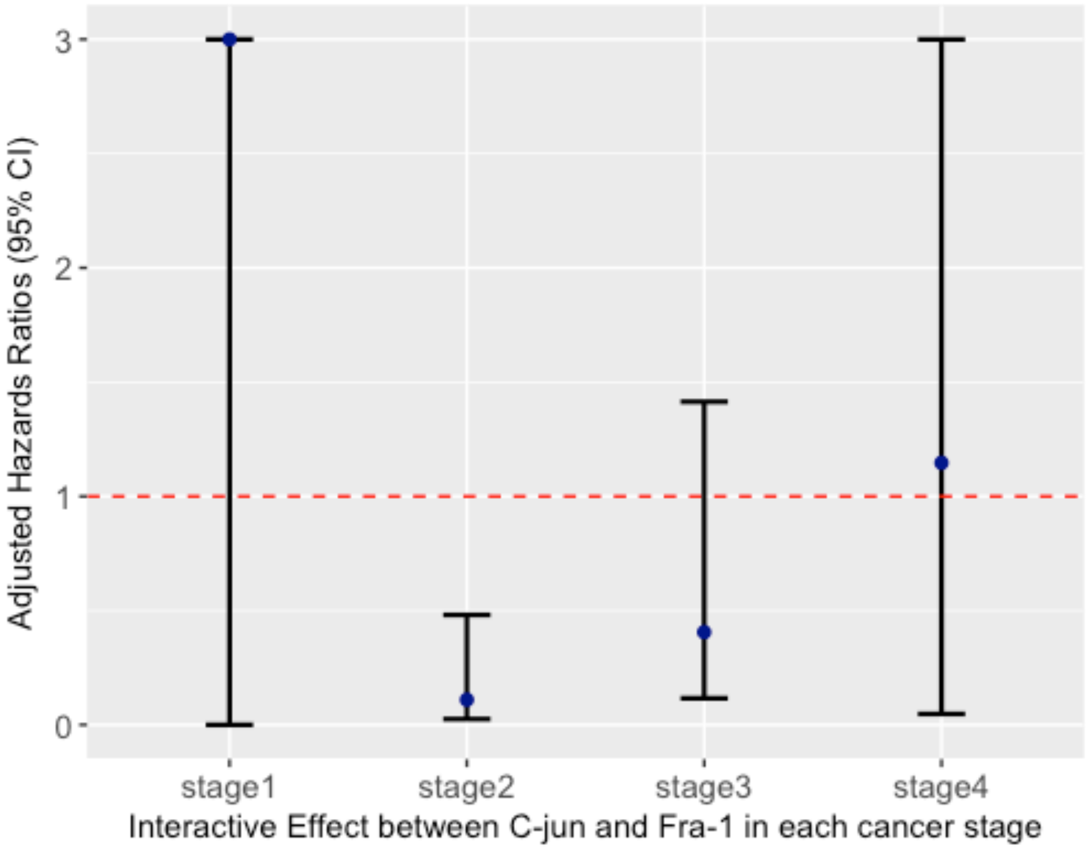

Supplement: Supplementary file 1 — Supplementary Figures [file 41598_2017_5106_MOESM1_ESM.pdf]
